# The effects of PERMA model-based positive psychological intervention on fear of disease progression and subjective well-being in patients after coronary artery bypass grafting: a retrospective cohort study

**DOI:** 10.3389/fcvm.2026.1784517

**Published:** 2026-05-28

**Authors:** Aimei Wu, Hong Liu, Tingting Fang, Peipei Fan, Chenchen Yin, Siwen Shen

**Affiliations:** 1 Department of Thoracic Surgery II, Affiliated Hospital of Jiangnan University, Wuxi, China; 2 Department of Cardiothoracic Surgery, General Hospital of the Eastern Theater Command, PLA, Nanjing, China; 3 Department of Cardiovascular Surgery, Affiliated Hospital of Jiangnan University, Wuxi, China

**Keywords:** cardiac rehabilitation, coronary artery bypass grafting, fear of disease progression, PERMA model, positive psychology, psychological intervention, subjective well-being

## Abstract

**Background:**

Patients undergoing coronary artery bypass grafting (CABG) frequently experience fear of disease progression (FoP) and diminished psychological well-being during the postoperative recovery period. The PERMA model, encompassing Positive emotion, Engagement, Relationships, Meaning, and Accomplishment, represents a comprehensive framework within positive psychology that may address these psychological challenges. However, the clinical effectiveness of PERMA-based interventions in cardiac surgery patients requires further empirical validation through retrospective analysis of real-world data.

**Methods:**

This retrospective cohort study analyzed medical records of 60 patients who underwent CABG at our institution between January 2023 and December 2025. Patients were categorized into a control group (*n* = 30) receiving conventional perioperative nursing care and an observation group (*n* = 30) receiving PERMA model-based psychological intervention in addition to standard care. Group allocation was determined retrospectively based on documented nursing care protocols, as patients treated by PERMA-trained nurses received the intervention while those treated by other nursing teams received standard care. Primary outcomes included FoP-Q-SF scores and PERMA-Profiler assessments. Secondary outcomes encompassed anxiety and depression scales, cardiac function parameters, and quality of life measures at three months post-discharge.

**Results:**

At three months post-intervention, the observation group demonstrated significantly lower FoP-Q-SF total scores compared to the control group (24.5 ± 4.8 vs. 31.2 ± 5.5, *P* < 0.001). All five PERMA dimensions showed statistically significant improvements in the intervention group (*P* < 0.05). Furthermore, the observation group exhibited superior cardiac rehabilitation outcomes, including enhanced left ventricular ejection fraction, greater six-minute walk distance, and improved rehabilitation adherence.

**Conclusion:**

PERMA model-based positive psychological intervention is associated with reduced fear of disease progression, enhanced subjective well-being, and improved postoperative recovery indicators in CABG patients. These findings support the integration of this intervention framework into cardiac surgery nursing protocols, though prospective randomized trials are needed to confirm causal relationships.

## 1 Introduction

Coronary artery disease remains the leading cause of mortality and morbidity worldwide, with coronary artery bypass grafting (CABG) serving as a fundamental revascularization strategy for patients with complex multivessel disease (1). While CABG surgery has demonstrated substantial efficacy in alleviating anginal symptoms and improving myocardial perfusion, the procedure itself constitutes a significant physiological and psychological stressor that extends well beyond the immediate postoperative period (2). The invasive nature of cardiac surgery, combined with the inherent vulnerability of having one's heart operated upon, creates a unique constellation of psychological challenges that profoundly influence recovery trajectories and long-term outcomes (3). Contemporary estimates suggest that approximately 30%–50% of patients undergoing CABG experience clinically significant levels of psychological distress during their recovery, manifesting as anxiety, depression, and pervasive fear regarding their cardiac condition (4). This psychological burden not only diminishes subjective quality of life but also exerts measurable negative effects on physiological healing processes, medication adherence, and engagement with cardiac rehabilitation programs (5). The recognition that psychological factors substantially influence cardiovascular outcomes has catalyzed increasing attention toward developing and implementing effective psychological interventions within cardiac care settings.

Fear of disease progression (FoP), alternatively conceptualized as fear of recurrence or fear of progression, represents a particularly salient psychological phenomenon among cardiac surgery patients that has garnered substantial research attention in recent years (6). This construct encompasses patients' apprehensions regarding potential bypass graft occlusion, disease advancement, functional deterioration, and ultimately, cardiac-related mortality (7). Unlike transient situational anxiety, FoP in cardiac patients often assumes a persistent, intrusive quality that colors daily functioning and decision-making processes long after surgical recovery has concluded (8). Research has established that elevated FoP is associated with maladaptive health behaviors, including avoidance of physical activity due to fear of cardiac strain, hypervigilance toward bodily sensations, and excessive healthcare utilization patterns (9). Furthermore, chronic activation of fear-related neural circuits and associated sympathetic nervous system arousal may itself contribute to adverse cardiovascular remodeling and increased arrhythmic risk (10). The cyclical relationship between psychological fear and physiological cardiac vulnerability underscores the critical importance of addressing FoP as an integral component of comprehensive cardiac rehabilitation.

Traditional approaches to perioperative nursing care in cardiac surgery have predominantly emphasized biomedical parameters, focusing on hemodynamic monitoring, wound management, infection prevention, and physical rehabilitation protocols (11). While these physiological dimensions of care remain indisputably essential, the exclusive concentration on biological metrics has created a notable gap in addressing the psychological architecture of recovery (12). Conventional patient education interventions typically provide disease information and self-care instructions in a didactic format that, while informative, fails to adequately engage the emotional and motivational dimensions of patient experience (13). This limitation is particularly consequential given the growing body of evidence demonstrating bidirectional relationships between psychological states and cardiovascular physiology. Systematic reviews have documented that psychological interventions can produce meaningful improvements in cardiac outcomes, yet the optimal theoretical frameworks and intervention modalities for this patient population remain subjects of ongoing investigation (14). The need for structured, theoretically grounded psychological intervention protocols that can be feasibly integrated into existing cardiac care pathways has become increasingly apparent to clinicians and researchers alike (3). Cardiac rehabilitation programs following CABG have been shown to improve health-related quality of life across both physical and mental domains, further supporting the need for comprehensive approaches that address psychological well-being alongside physical recovery (15, 16).

The PERMA model, articulated by Martin Seligman as a foundational framework within positive psychology, offers a comprehensive theoretical architecture for conceptualizing and promoting human flourishing (17). This model identifies five empirically validated pillars of psychological well-being: Positive emotion (P), which encompasses experiences of joy, gratitude, contentment, and optimism; Engagement (E), referring to states of flow and absorption in meaningful activities; Relationships (R), capturing the quality and depth of social connections; Meaning (M), addressing the sense of purpose and significance derived from life pursuits; and Accomplishment (A), reflecting the satisfaction associated with goal achievement and mastery (18). Unlike deficit-focused psychological models that primarily target symptom reduction, the PERMA framework emphasizes the cultivation of positive psychological resources that may serve protective and promotive functions (19). This strengths-based orientation aligns particularly well with rehabilitation contexts, where the goal extends beyond mere symptom amelioration toward optimal functioning and enhanced quality of life. The PERMA model has demonstrated applicability across diverse clinical populations, including patients with chronic medical conditions, though its specific efficacy in cardiac surgery populations has received comparatively limited empirical attention (20).

The application of positive psychological interventions to cardiac populations represents an emerging frontier with considerable theoretical and practical promise (21). Preliminary research suggests that interventions targeting positive affect, social connection, and meaning-making may produce beneficial effects on both psychological well-being and cardiovascular parameters (22). The PERMA framework provides a comprehensive structure for intervention development that addresses multiple dimensions of psychological functioning simultaneously, potentially generating synergistic effects that exceed those achievable through more narrowly focused approaches (23). Furthermore, the emphasis on building positive resources rather than merely reducing negative symptoms may be particularly relevant for cardiac patients, for whom maintaining motivation and hope throughout prolonged recovery periods is essential (24). However, the translation of PERMA-based interventions into clinical cardiac nursing practice remains in nascent stages, with limited empirical data available to guide implementation decisions and evaluate effectiveness in real-world healthcare settings (25). The retrospective examination of clinical outcomes among patients who have received PERMA-informed care offers a valuable opportunity to assess the practical utility and effectiveness of this approach under naturalistic conditions.

The present investigation was designed to address this knowledge gap through a retrospective cohort analysis examining the associations between PERMA model-based positive psychological intervention and fear of disease progression and subjective well-being among patients recovering from CABG surgery (26). By analyzing clinical records and outcome data collected over a three-year period at a single cardiac surgery center, this study aims to evaluate whether patients who received PERMA-informed psychological care demonstrated superior psychological and physiological outcomes compared to those receiving conventional nursing care (27). Specifically, this research hypothesizes that PERMA-based intervention will be associated with reduced fear of disease progression, enhanced subjective well-being across all five PERMA dimensions, improved cardiac functional recovery, greater behavioral adherence to treatment recommendations, and superior overall quality of life (28). Secondary objectives include exploring associations between psychological improvements and physiological outcomes to generate hypotheses regarding potential mechanisms through which positive psychological intervention may influence cardiac recovery. The findings of this investigation are anticipated to provide empirical guidance for the integration of positive psychology principles into cardiac surgical nursing care and to contribute to the growing evidence base supporting comprehensive psychocardiac rehabilitation approaches (29).

## 2 Materials and methods

### 2.1 Study design and participants

This investigation employed a single-center retrospective cohort design to evaluate the associations between PERMA model-based positive psychological intervention and clinical outcomes in patients following coronary artery bypass grafting surgery. The study period extended from January 1, 2023, through December 31, 2025, encompassing three years of clinical data collection at the Department of Cardiac Surgery in our tertiary care institution. Medical records of patients who underwent CABG during this period were systematically reviewed to identify eligible participants and extract relevant outcome data. The retrospective cohort design was selected as it permitted evaluation of intervention associations under naturalistic clinical conditions and avoided the ethical constraints associated with withholding potentially beneficial psychological care from control participants in a prospective randomized trial. This study involved the analysis of clinical data collected from human subjects during routine medical care.

**Figure 1:**
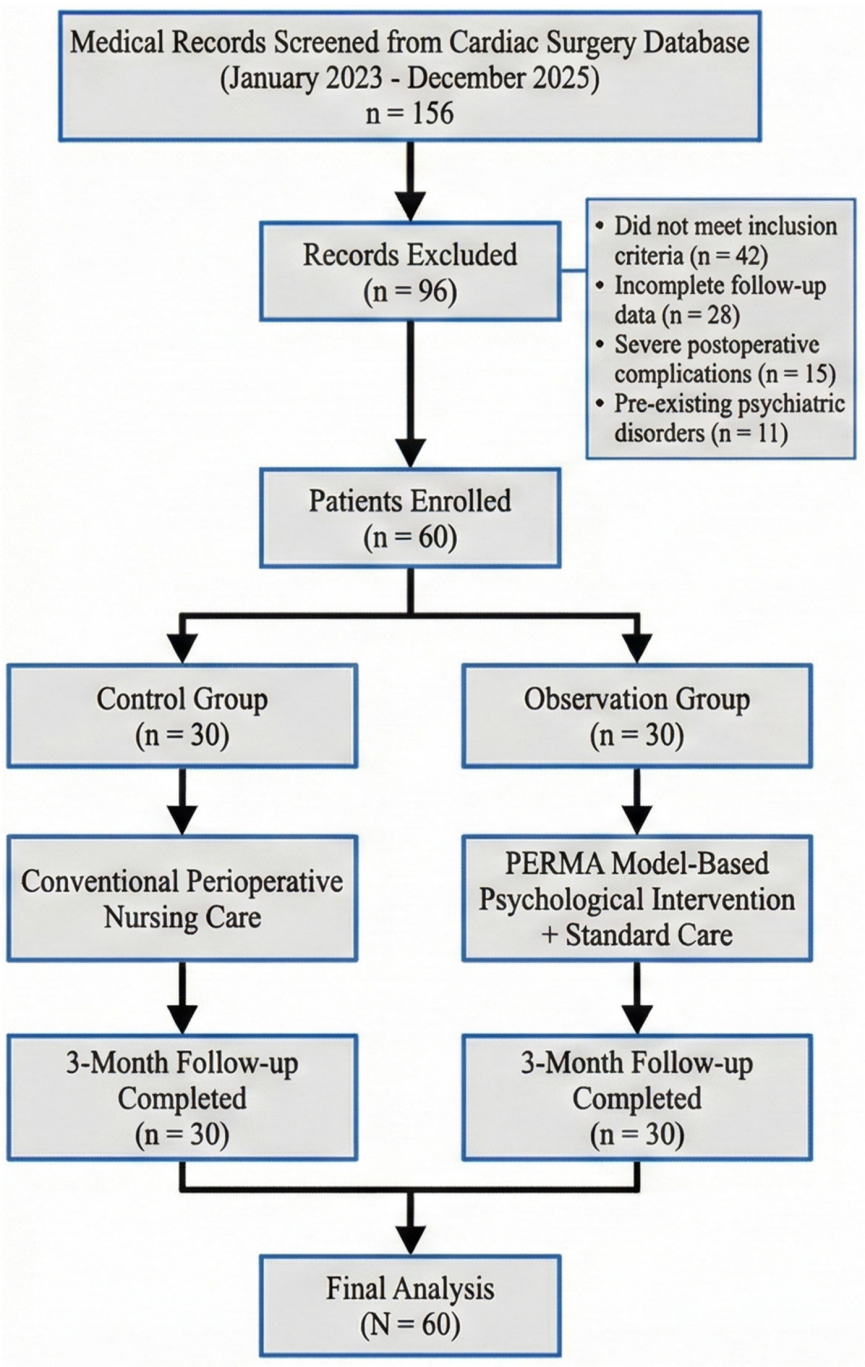
Flowchart of participant screening, enrollment, and group allocation in the retrospective cohort study.

Patient inclusion criteria comprised the following requirements: confirmed diagnosis of coronary artery disease with indication for surgical revascularization; first-time CABG procedure without concomitant valvular or other cardiac surgery; age between 45 and 75 years; preoperative New York Heart Association (NYHA) functional classification of II or III; and availability of complete medical records including psychological assessments at baseline (admission), one month post-discharge, and three months post-discharge. The age range of 45 to 75 years was selected based on several clinical and methodological considerations. Patients younger than 45 years undergoing CABG represent an atypically young population with distinct pathophysiological profiles, often associated with familial hypercholesterolemia or accelerated atherosclerosis, whose psychological responses and recovery trajectories may differ substantially from the typical CABG population (1). Patients older than 75 years frequently present with multiple age-related comorbidities, cognitive changes, and frailty syndromes that may confound the assessment of psychological intervention effects and compromise the validity of self-report instruments (2). Thus, the 45–75 age range was chosen to capture the most representative CABG population while minimizing the influence of age-related confounders on psychological outcome measures. Exclusion criteria included: pre-existing psychiatric disorders requiring pharmacological treatment; cognitive impairment preventing valid completion of psychological assessment instruments; postoperative complications resulting in prolonged intensive care unit stay exceeding seven days or requiring reoperation; severe comorbidities with life expectancy less than one year; and incomplete follow-up data precluding outcome assessment. Application of these criteria yielded a final sample of 60 patients with complete data available for analysis (Figure 1).

### 2.2 Group allocation

Patients were retrospectively assigned to study groups based on the documented nursing care protocols received during their hospitalization and follow-up period. During the study period, our institution implemented a staged introduction of the PERMA model-based psychological intervention as part of a quality improvement initiative in cardiac nursing care. Specifically, a cohort of cardiac rehabilitation nurses (*n* = 5) underwent a structured 40-hour training program in PERMA-based psychological intervention techniques conducted by a certified positive psychology practitioner between October and December 2022. Following training completion and competency assessment, these nurses began implementing the PERMA intervention with their assigned patients beginning in January 2023. Patients were assigned to nursing teams based on standard ward scheduling and bed allocation procedures, which operated independently of patient clinical characteristics or preferences, thereby approximating a quasi-random allocation mechanism. The control group (*n* = 30) comprised patients whose medical records indicated receipt of conventional perioperative cardiac surgery nursing care, including standard health education regarding disease management, medication guidance, activity recommendations, and routine rehabilitation training without documented implementation of structured psychological intervention components. The observation group (*n* = 30) included patients whose nursing records documented implementation of PERMA model-based psychological intervention in addition to standard care. Identification of observation group patients required documentation of specific PERMA intervention elements, including gratitude journaling activities, personal strengths exploration, social support group participation, meaning-reconstruction discussions, and progressive goal-setting for rehabilitation milestones. This documentation-based allocation strategy ensured that group membership reflected actual clinical care received rather than intended treatment assignment.

### 2.3 Intervention description

The PERMA model-based intervention was implemented by five trained cardiac rehabilitation nurses who had completed a 40-hour certification training program encompassing theoretical foundations of the PERMA model, practical intervention delivery skills, standardized session protocols, and supervised practice sessions with simulated patients. All training nurses possessed a minimum of three years of cardiac nursing experience and held bachelor's degrees in nursing. Intervention fidelity was monitored through structured nursing documentation templates that required recording of specific PERMA activities delivered during each patient encounter, weekly case review meetings among the trained nursing team, and periodic supervision by the positive psychology trainer during the first six months of implementation.

The intervention was delivered across three phases: the in-hospital phase (from postoperative day 2 through discharge, typically 7–10 days), a transitional phase (weeks 1–4 post-discharge, delivered via weekly telephone or video sessions lasting 30–45 min each), and a maintenance phase (months 2–3 post-discharge, delivered via biweekly telephone sessions lasting 20–30 min each). In total, each patient in the observation group received approximately 8–12 structured PERMA intervention sessions over the three-month follow-up period, in addition to daily brief bedside interactions during hospitalization.

The Positive emotion (P) component involved guided documentation of “three good things” that occurred each day, cultivation of gratitude through reflection exercises, and instruction in positive reframing techniques for challenging recovery experiences. The Engagement (E) dimension was addressed through facilitation of participation in cardiac rehabilitation exercises, introduction to mindfulness meditation practices, and identification of personally meaningful activities that promoted absorption and flow states. The Relationships (R) component encompassed enhancement of family member involvement in care processes, organization of peer support group meetings with other cardiac surgery patients held biweekly during hospitalization, and strengthening of therapeutic relationships with healthcare providers. The Meaning (M) dimension involved structured interviews exploring patients' sources of life meaning and purpose, facilitation of life narrative reconstruction integrating the cardiac illness experience, and identification of values-congruent goals for the post-recovery period. Finally, the Accomplishment (A) component comprised establishment of progressive, achievable rehabilitation goals including targets for six-minute walk distance, and systematic documentation of goal attainment to reinforce mastery experiences. Standard care in both groups included conventional health education, medication administration and guidance, wound care, activity progression protocols, and discharge planning.

### 2.4 Assessment instruments

Fear of disease progression was assessed using the Fear of Progression Questionnaire-Short Form (FoP-Q-SF), a validated 12-item instrument measuring anxiety specifically related to disease progression and recurrence. Each item is rated on a 5-point Likert scale from 1 (never) to 5 (very often), yielding total scores ranging from 12 to 60, with higher scores indicating greater fear of progression. The instrument comprises two subscales: physiological health concerns and social-family concerns. The FoP-Q-SF has demonstrated satisfactory psychometric properties in cardiac populations, with Cronbach's alpha coefficients exceeding 0.85 in validation studies. The Chinese version of the FoP-Q-SF was translated and validated by Wu et al. (30), demonstrating adequate construct validity and internal consistency (Cronbach's α = 0.88) in Chinese patients with chronic diseases. In the present sample, internal consistency was excellent (Cronbach's α = 0.89).

Subjective well-being was measured using the PERMA-Profiler, a 23-item questionnaire assessing the five dimensions of the PERMA model along with negative emotion and overall health satisfaction. Items are rated on an 11-point scale from 0 to 10, with higher scores reflecting greater well-being on positive dimensions and lower well-being on the negative emotion subscale. The instrument provides subscale scores for Positive emotion, Engagement, Relationships, Meaning, Accomplishment, Negative emotion, Health, and Overall well-being. The Chinese version of the PERMA-Profiler was validated by Li et al. (31), confirming five-factor structure and demonstrating good reliability (Cronbach's α ranging from 0.78 to 0.90 across subscales) in Chinese adult populations. In the current study, Cronbach's alpha values ranged from 0.81 to 0.91 across subscales.

Anxiety and depression were assessed using the Self-Rating Anxiety Scale (SAS) and Self-Rating Depression Scale (SDS), respectively. Both instruments contain 20 items rated on a 4-point scale, with raw scores converted to standardized scores ranging from 25 to 100. Standardized scores exceeding 50 indicate clinically significant symptoms. The Chinese versions of the SAS and SDS have been extensively validated and are widely used in Chinese clinical research, with established normative data for Chinese populations (32). Both scales have demonstrated satisfactory validity in Chinese cardiac patient populations with reported Cronbach's alpha values above 0.80. Quality of life was evaluated using the Medical Outcomes Study 36-Item Short Form Health Survey (SF-36). The Chinese version of the SF-36 was translated and validated by Li et al. (33), demonstrating satisfactory reliability (Cronbach's α ranging from 0.72 to 0.88 across domains) and construct validity in Chinese populations. The SF-36 assesses eight health domains: physical functioning, role limitations due to physical health, bodily pain, general health perceptions, vitality, social functioning, role limitations due to emotional problems, and mental health. Domain scores range from 0 to 100, with higher scores indicating better health status.

### 2.5 Physiological measures

Cardiac function was evaluated through transthoracic echocardiography with measurement of left ventricular ejection fraction (LVEF). Functional exercise capacity was assessed using the six-minute walk test (6 MWT), measuring the maximum distance walked on a flat corridor during six minutes according to standardized protocols. B-type natriuretic peptide (BNP) levels were measured from venous blood samples as a biomarker of cardiac stress and ventricular function. Resting heart rate was recorded during routine vital sign assessment. All physiological measurements were obtained at baseline and at three months post-discharge.

### 2.6 Behavioral adherence assessment

Exercise adherence was defined as participation in structured cardiac rehabilitation exercise sessions at a frequency of at least three times per week for the duration of the follow-up period, as documented in cardiac rehabilitation attendance records and corroborated by patient self-report during follow-up telephone assessments. Dietary adherence was defined as self-reported compliance with prescribed low-sodium (<6 g/day) and low-fat dietary recommendations, assessed through a standardized dietary adherence questionnaire administered by nursing staff at the three-month follow-up visit. Patients who reported adherence to dietary recommendations on at least 80% of days during the follow-up period were classified as adherent.

### 2.7 Ethical considerations

This retrospective study was approved by the Institutional Review Board of Affiliated Hospital of Jiangnan University. As this investigation involved the analysis of clinical data collected from human subjects during routine medical care, it was conducted under full ethical oversight. Given the retrospective nature of the investigation utilizing de-identified medical record data, the requirement for individual written informed consent for research participation was waived by the ethics committee in accordance with applicable regulations. However, all patients had provided general informed consent for the use of their de-identified clinical data for quality improvement and research purposes at the time of hospital admission, as part of our institution's standard admission procedures. All data were handled in accordance with institutional policies for protection of patient confidentiality. The study was conducted in compliance with the Declaration of Helsinki and applicable local regulations governing research involving human subjects.

### 2.8 Statistical analysis

All statistical analyses were performed using IBM SPSS Statistics version 26.0 (IBM Corporation, Armonk, NY, USA). Continuous variables were assessed for normality of distribution using the Kolmogorov–Smirnov test and are presented as mean ± standard deviation (SD). Categorical variables are expressed as frequencies and percentages. Baseline comparisons between groups employed independent samples t-tests for continuous variables and chi-square tests for categorical variables. Changes in outcome measures over time were analyzed using repeated measures analysis of variance (ANOVA) with group as the between-subjects factor and time as the within-subjects factor. *Post-hoc* pairwise comparisons were conducted using Bonferroni correction for multiple comparisons. To address potential confounding in the non-randomized design, multivariable linear regression models were constructed for primary outcomes (FoP-Q-SF total score at three months and PERMA overall well-being score at three months), adjusting for baseline values, age, sex, education level, NYHA class, and number of grafted vessels. Propensity score analysis was performed using logistic regression to estimate each patient's probability of receiving the PERMA intervention based on available baseline covariates (age, sex, BMI, education, marital status, hypertension, diabetes, hyperlipidemia, NYHA class, CPB duration, number of grafted vessels, and ICU length of stay). The resulting propensity scores were then included as a covariate in sensitivity analyses to further assess the robustness of between-group comparisons. Pearson correlation coefficients were calculated to examine relationships between psychological and physiological outcome variables. Effect sizes were computed using Cohen's d for between-group comparisons, and 95% confidence intervals (CIs) for between-group mean differences were reported for primary outcomes. Given the multiple endpoints tested, results should be interpreted with appropriate caution regarding multiplicity, and the primary endpoints (FoP-Q-SF and PERMA overall well-being) should be prioritized in interpretation. A two-tailed P-value less than 0.05 was considered statistically significant for all analyses.

## 3 Results

### 3.1 Baseline demographic and clinical characteristics

As presented in Table 1, the two study groups demonstrated comparable baseline demographic and clinical characteristics, confirming the adequacy of retrospective group allocation for between-group comparisons. The mean age of participants was 64.2 ± 5.8 years in the observation group and 63.8 ± 6.1 years in the control group (*t* = 0.261, *P* = 0.795). Sex distribution was similar between groups, with males comprising 60% and 63.3% of the observation and control groups, respectively (*χ*^2^ = 0.069, *P* = 0.792). Body mass index, educational attainment, and marital status did not differ significantly between groups. Regarding cardiovascular comorbidities, hypertension was present in 66.7% of observation group patients and 70.0% of control group patients (*χ*^2^ = 0.077, *P* = 0.782). Type 2 diabetes mellitus prevalence was 40.0% versus 36.7% (*χ*^2^ = 0.070, *P* = 0.791), and hyperlipidemia was documented in 50.0% versus 53.3% of patients (*χ*^2^ = 0.067, *P* = 0.796). Preoperative NYHA functional class distribution, cardiopulmonary bypass duration, number of grafted vessels, and intensive care unit length of stay were also statistically equivalent between groups, indicating that both cohorts experienced comparable surgical complexity and immediate postoperative courses. The estimated propensity scores did not differ significantly between groups (observation: 0.52 ± 0.14 vs. control: 0.48 ± 0.13; *t* = 1.14, *P* = 0.259), further supporting the comparability of the two cohorts on measured baseline characteristics.

**Table 1:** Baseline demographic and clinical characteristics of study participants.

Characteristics	Observation group (*n* = 30)	Control group (*n* = 30)	t/*χ*²	*P*-value
Age (years, Mean ± SD)	64.2 ± 5.8	63.8 ± 6.1	0.261	0.795
Sex (Male/Female)	18/12	19/11	0.069	0.792
BMI (kg/m²)	24.5 ± 3.1	24.8 ± 2.9	−0.387	0.700
Education (years)	10.5 ± 2.4	10.1 ± 2.6	0.619	0.538
Married, n (%)	28 (93.3%)	27 (90.0%)	0.218	0.640
Hypertension, n (%)	20 (66.7%)	21 (70.0%)	0.077	0.782
Type 2 Diabetes, n (%)	12 (40.0%)	11 (36.7%)	0.070	0.791
Hyperlipidemia, n (%)	15 (50.0%)	16 (53.3%)	0.067	0.796
NYHA Class (II/III)	18/12	17/13	0.067	0.796
CPB Duration (min)	88.5 ± 15.2	89.2 ± 14.8	−0.181	0.857
Grafted Vessels (n)	3.1 ± 0.8	3.0 ± 0.7	0.513	0.610
ICU Stay (hours)	48.5 ± 12.4	49.1 ± 13.5	−0.179	0.859

BMI, body mass index; NYHA, New York heart association; CPB, cardiopulmonary bypass; ICU, intensive care unit.

### 3.2 Fear of disease progression and negative affect

Analysis of fear of disease progression revealed substantial between-group differences favoring the PERMA intervention, as detailed in Table 2. At baseline (T0), FoP-Q-SF total scores were comparable between the observation group (38.5 ± 6.2) and control group (39.1 ± 5.8; *t* = −0.387, *P* = 0.700), indicating equivalent levels of disease-related fear prior to intervention differentiation. However, by one month post-discharge (T1), the observation group demonstrated significantly lower FoP scores (30.2 ± 5.4) compared to controls (35.6 ± 6.1; *t* = −3.628, *P* < 0.001), representing a clinically meaningful reduction. This between-group difference was further amplified at three months post-discharge (T2), with observation group scores of 24.5 ± 4.8 versus control group scores of 31.2 ± 5.5 (*t* = −5.024, *P* < 0.001; Cohen's d = 1.30; 95% CI for mean difference: −9.37 to −4.03). Repeated measures ANOVA confirmed a significant group-by-time interaction effect (*F* = 18.42, *P* < 0.001), indicating that the intervention was associated with differential trajectories of change across the follow-up period. In the multivariable linear regression model adjusting for baseline FoP-Q-SF score, age, sex, education, NYHA class, and number of grafted vessels, group allocation remained a significant predictor of three-month FoP-Q-SF scores (*β* = −5.82, 95% CI: −8.41 to −3.23, *P* < 0.001). Propensity score-adjusted analysis yielded consistent results (*β* = −5.65, 95% CI: −8.30 to −3.00, *P* < 0.001).

**Table 2:** Comparison of fear of disease progression and psychological distress scores.

Measure	Time point	Observation (*n* = 30)	Control (*n* = 30)	*t*-value	*P*-value
FoP-Q-SF Total	T0 (Baseline)	38.5 ± 6.2	39.1 ± 5.8	−0.387	0.700
(Range: 12–60)	T1 (1 Month)	30.2 ± 5.4	35.6 ± 6.1	−3.628	<0.001
	T2 (3 Months)	24.5 ± 4.8	31.2 ± 5.5	−5.024	<0.001
FoP-Physiological	T2 (3 Months)	12.1 ± 2.3	16.5 ± 3.1	−6.230	<0.001
FoP-Social/Family	T2 (3 Months)	12.4 ± 2.5	14.7 ± 2.8	−3.354	0.001
SAS Score	T2 (3 Months)	42.5 ± 5.6	48.9 ± 6.2	−4.195	<0.001
SDS Score	T2 (3 Months)	43.1 ± 5.9	49.5 ± 6.5	−3.991	<0.001

FoP-Q-SF, fear of progression questionnaire-short form; SAS, self-rating anxiety scale; SDS, self-rating depression scale.

**Figure 2:**
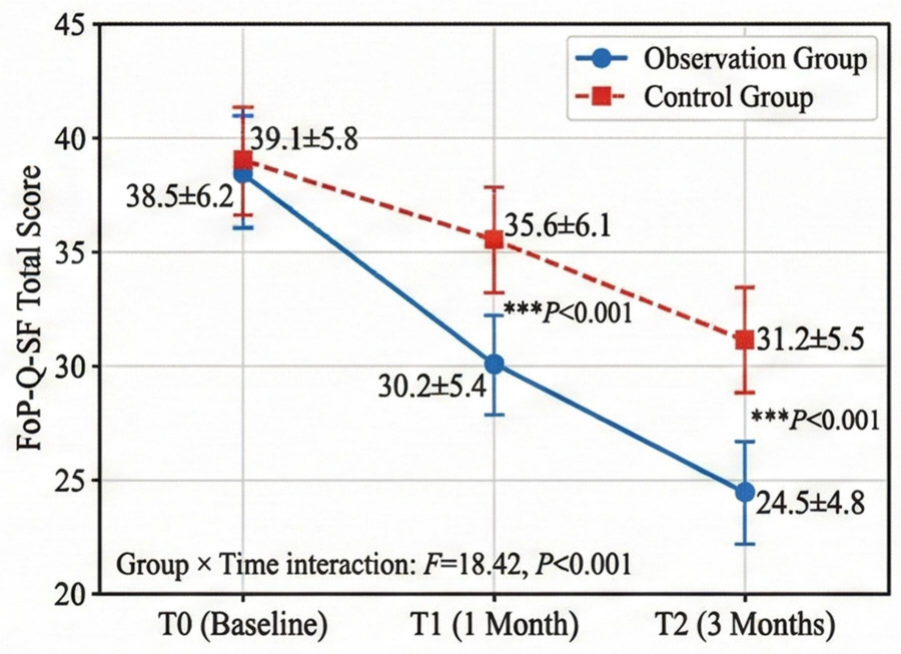
Temporal trajectory of fear of disease progression (FoP-Q-SF) total scores across assessment timepoints. The observation group (solid line) demonstrates progressive reduction compared to the control group (dashed line).

Examination of FoP subscale scores at three months revealed that both dimensions showed significant differences favoring the intervention group. The physiological health concerns subscale demonstrated particularly pronounced differences, with scores of 12.1 ± 2.3 in the observation group versus 16.5 ± 3.1 in controls (*t* = −6.230, *P* < 0.001; Cohen's d = 1.61). The social-family concerns subscale also favored the intervention group (12.4 ± 2.5 vs. 14.7 ± 2.8; t = −3.354, *P* = 0.001; Cohen's d = 0.87). These findings indicate that PERMA-based intervention addressed both health-related and interpersonal dimensions of disease progression fear. Corresponding improvements were observed in general psychological distress measures, with the observation group demonstrating significantly lower SAS anxiety scores (42.5 ± 5.6 vs. 48.9 ± 6.2; *t* = −4.195, *P* < 0.001; Cohen's d = 1.08) and SDS depression scores (43.1 ± 5.9 vs. 49.5 ± 6.5; *t* = −3.991, *P* < 0.001; Cohen's d = 1.03) at three-month follow-up. The temporal trajectory of FoP score changes across assessment timepoints is illustrated in Figure 2, demonstrating the progressive divergence between groups following intervention implementation.

### 3.3 Perma well-being profile analysis

As displayed in Table 3, comprehensive assessment of subjective well-being using the PERMA-Profiler revealed significant advantages for the intervention group across all evaluated dimensions. At three months post-discharge, the observation group demonstrated superior positive emotion scores (7.8 ± 1.2 vs. 6.1 ± 1.5; *t* = 4.835, *P* < 0.001; Cohen's d = 1.25), indicating enhanced experiences of joy, gratitude, and contentment. Engagement scores reflecting absorption in meaningful activities were also significantly higher in the observation group (7.2 ± 1.4 vs. 5.8 ± 1.6; *t* = 3.606, *P* < 0.001; Cohen's d = 0.93). The relationships dimension showed the most pronounced between-group difference (8.1 ± 1.1 vs. 6.5 ± 1.4; *t* = 4.908, *P* < 0.001; Cohen's d = 1.27), suggesting that intervention components targeting social support substantially strengthened patients' interpersonal connections.

**Table 3:** PERMA-Profiler dimension scores at three months post-discharge.

PERMA dimension	Observation (*n* = 30)	Control (*n* = 30)	*t*-value	*P*-value
Positive Emotion (P)	7.8 ± 1.2	6.1 ± 1.5	4.835	<0.001
Engagement (E)	7.2 ± 1.4	5.8 ± 1.6	3.606	<0.001
Relationships (R)	8.1 ± 1.1	6.5 ± 1.4	4.908	<0.001
Meaning (M)	7.5 ± 1.3	6.0 ± 1.5	4.139	<0.001
Accomplishment (A)	7.6 ± 1.2	6.2 ± 1.4	4.156	<0.001
Overall Well-being	7.9 ± 1.0	6.3 ± 1.2	5.602	<0.001
Negative Emotion	3.2 ± 1.1	5.4 ± 1.5	−6.480	<0.001
Health Satisfaction	7.5 ± 1.3	5.9 ± 1.6	4.251	<0.001

All dimensions scored on 0–10 scale. Higher scores indicate greater well-being except for negative emotion.

**Figure 3:**
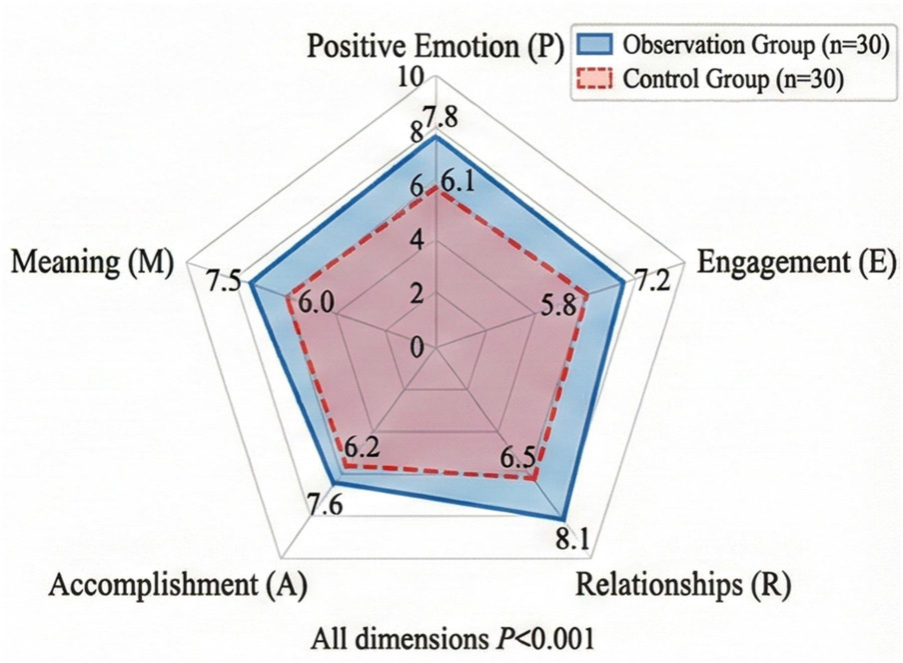
Radar chart comparison of pERMA five-dimension scores between observation and control groups at three months post-discharge.

Meaning dimension scores, reflecting perceived purpose and significance in life, were significantly elevated in the observation group (7.5 ± 1.3 vs. 6.0 ± 1.5; *t* = 4.139, *P* < 0.001; Cohen's d = 1.07). Accomplishment scores demonstrated similar patterns (7.6 ± 1.2 vs. 6.2 ± 1.4; *t* = 4.156, *P* < 0.001; Cohen's d = 1.07), indicating that systematic goal-setting and mastery experiences enhanced patients' sense of achievement. The overall well-being composite score was substantially higher in the intervention group (7.9 ± 1.0 vs. 6.3 ± 1.2; *t* = 5.602, *P* < 0.001; Cohen's d = 1.45; 95% CI for mean difference: 1.03 to 2.17). In the multivariable regression model, group allocation remained a significant predictor of overall well-being at three months after covariate adjustment (*β* = 1.42, 95% CI: 0.88 to 1.96, *P* < 0.001). Propensity score-adjusted analysis confirmed this association (*β* = 1.38, 95% CI: 0.82 to 1.94, *P* < 0.001). Complementing these improvements in positive psychological dimensions, the observation group reported significantly lower negative emotion scores (3.2 ± 1.1 vs. 5.4 ± 1.5; *t* = −6.480, *P* < 0.001; Cohen's d = 1.67) and greater health satisfaction (7.5 ± 1.3 vs. 5.9 ± 1.6; *t* = 4.251, *P* < 0.001; Cohen's d = 1.10). Figure 3 presents a radar chart visualization of PERMA dimension scores, illustrating the comprehensive advantage of the intervention group across all five pillars of psychological flourishing.

### 3.4 Cardiac function and behavioral adherence

Table 4 presents cardiac function parameters and behavioral adherence outcomes at three months post-discharge. The observation group demonstrated significantly higher left ventricular ejection fraction (58.4 ± 4.5% vs. 55.2 ± 5.1%; *t* = 2.576, *P* = 0.012; Cohen's d = 0.67), suggesting enhanced myocardial recovery. The six-minute walk distance showed pronounced between-group differences (425.6 ± 38.2 m vs. 380.5 ± 42.1 m; t = 4.341, *P* < 0.001; Cohen's d = 1.12), indicating superior functional exercise capacity in the intervention group. BNP levels were significantly lower in the observation group (125.4 ± 45.2 pg/mL vs. 188.6 ± 52.4 pg/mL; *t* = −4.992, *P* < 0.001; Cohen's d = 1.29), reflecting reduced cardiac stress. Resting heart rate was also more favorable in the intervention group (72.5 ± 6.4 vs. 78.2 ± 7.1 bpm; t = −3.265, *P* = 0.002; Cohen's d = 0.84).

**Table 4:** Cardiac function parameters and behavioral adherence at three months.

Parameter	Observation (*n* = 30)	Control (*n* = 30)	t/χ²	*P*-value
LVEF (%)	58.4 ± 4.5	55.2 ± 5.1	2.576	0.012
6 MWT Distance (m)	425.6 ± 38.2	380.5 ± 42.1	4.341	<0.001
BNP (pg/mL)	125.4 ± 45.2	188.6 ± 52.4	−4.992	<0.001
Resting Heart Rate (bpm)	72.5 ± 6.4	78.2 ± 7.1	−3.265	0.002
Exercise Adherence, n (%)	26 (86.7%)	18 (60.0%)	5.455	0.019
Dietary Adherence, n (%)	27 (90.0%)	20 (66.7%)	4.812	0.028

LVEF, left ventricular ejection fraction; 6MWT, six-minute walk test; BNP, B-type natriuretic peptide.

Behavioral adherence metrics revealed substantial advantages for the intervention group in rehabilitation and dietary domains. Cardiac rehabilitation exercise adherence, defined as attending structured rehabilitation exercise sessions at least three times per week as documented in rehabilitation attendance records, was achieved by 86.7% of intervention patients versus 60.0% of controls (*χ*^2^ = 5.455, *P* = 0.019). Dietary adherence to low-sodium, low-fat recommendations, assessed through standardized dietary adherence questionnaires at three-month follow-up, was documented in 90.0% of the observation group compared to 66.7% of controls (*χ*^2^ = 4.812, *P* = 0.028). These adherence differences suggest that psychological well-being improvements may have translated into enhanced self-management behaviors.

### 3.5 Health-related quality of life

Table 5 presents SF-36 quality of life domain scores at three months post-discharge. The observation group demonstrated significantly higher scores across seven of eight domains. Physical functioning scores were substantially elevated in the intervention group (82.5 ± 10.2 vs. 70.4 ± 12.5; *t* = 4.108, *P* < 0.001), as were role-physical scores (75.5 ± 15.6 vs. 60.2 ± 18.4; *t* = 3.476, *P* = 0.001). General health perceptions showed pronounced differences (78.4 ± 11.2 vs. 62.5 ± 10.8; *t* = 5.597, *P* < 0.001). Vitality scores were significantly higher in the observation group (72.5 ± 10.5 vs. 58.4 ± 11.2; *t* = 5.030, *P* < 0.001), indicating greater energy and reduced fatigue. Social functioning (85.2 ± 12.4 vs. 71.5 ± 14.2; *t* = 3.978, *P* < 0.001) and role-emotional (78.5 ± 16.2 vs. 62.4 ± 18.5; *t* = 3.587, *P* = 0.001) domains also favored the intervention group. Mental health scores showed the largest between-group effect (81.2 ± 9.8 vs. 65.4 ± 10.5; *t* = 6.027, *P* < 0.001). Only bodily pain scores did not differ significantly between groups (80.2 ± 12.4 vs. 76.5 ± 13.1; *t* = 1.124, *P* = 0.266).

**Table 5:** SF-36 quality of life domain scores at three months post-discharge.

SF-36 domain (0–100)	Observation (*n* = 30)	Control (*n* = 30)	*t*-value	*P*-value
Physical Functioning (PF)	82.5 ± 10.2	70.4 ± 12.5	4.108	<0.001
Role-Physical (RP)	75.5 ± 15.6	60.2 ± 18.4	3.476	0.001
Bodily Pain (BP)	80.2 ± 12.4	76.5 ± 13.1	1.124	0.266
General Health (GH)	78.4 ± 11.2	62.5 ± 10.8	5.597	<0.001
Vitality (VT)	72.5 ± 10.5	58.4 ± 11.2	5.030	<0.001
Social Functioning (SF)	85.2 ± 12.4	71.5 ± 14.2	3.978	<0.001
Role-Emotional (RE)	78.5 ± 16.2	62.4 ± 18.5	3.587	0.001
Mental Health (MH)	81.2 ± 9.8	65.4 ± 10.5	6.027	<0.001

Higher scores indicate better health status across all domains.

**Figure 4:**
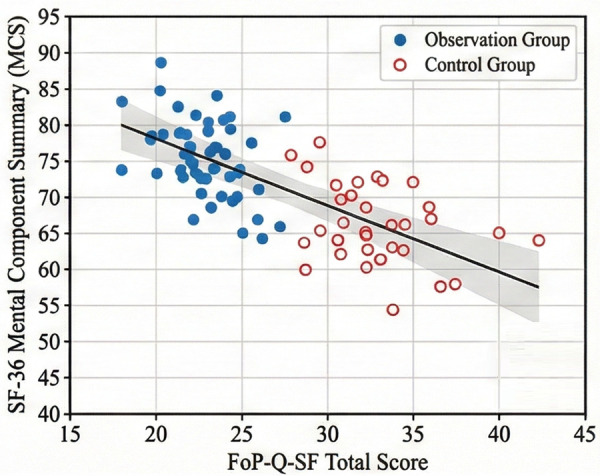
Scatter plot depicting the correlation between fear of disease progression (FoP-Q-SF) scores and SF-36 mental component summary scores (*r* = −0.68, *P* < 0.01).

Correlation analysis examining relationships between primary psychological outcomes and quality of life revealed significant associations. FoP-Q-SF total scores demonstrated strong negative correlations with SF-36 physical component summary (*r* = −0.58, *P* < 0.01) and mental component summary scores (*r* = −0.68, *P* < 0.01), indicating that greater fear of disease progression was associated with diminished quality of life. Conversely, PERMA overall well-being scores correlated positively with both physical (*r* = 0.65, *P* < 0.01) and mental (*r* = 0.72, *P* < 0.01) quality of life components. These correlational findings, illustrated in Figure 4, support the hypothesized pathway through which psychological intervention may influence functional outcomes.

## 4 Discussion

This retrospective cohort study provides evidence that PERMA model-based positive psychological intervention is associated with reduced fear of disease progression and enhanced subjective well-being in patients following coronary artery bypass grafting surgery. The observation group receiving PERMA-informed care demonstrated significantly lower fear of progression scores, superior well-being across all five PERMA dimensions, improved cardiac functional parameters, enhanced behavioral adherence, and better health-related quality of life compared to patients receiving conventional nursing care alone. These findings align with theoretical predictions regarding the protective and promotive functions of positive psychological resources in medical populations and extend the empirical foundation supporting integration of structured psychological interventions into cardiac rehabilitation protocols (34, 35).

The substantial reduction in fear of disease progression observed in the intervention group represents a clinically meaningful finding with important implications for patient welfare and recovery trajectories. Fear of disease progression constitutes a significant psychological burden for cardiac surgery patients that extends beyond general anxiety to encompass specific concerns regarding graft patency, disease advancement, and cardiac mortality (7). Our findings indicating that PERMA-based intervention was associated with significantly reduced both physiological health concerns and social-family concerns dimensions of FoP suggest that this comprehensive approach addresses the multidimensional nature of disease-related fear. The large effect sizes observed (Cohen's d = 1.30 for FoP-Q-SF total score) and the consistency of results across unadjusted, multivariable-adjusted, and propensity score-adjusted analyses strengthen confidence in the robustness of these associations. These results are consistent with prior research demonstrating that positive psychological interventions can effectively reduce distress in chronic disease populations, though few studies have specifically examined FoP as an outcome variable in cardiac surgery contexts (20). The cultivation of positive emotions through gratitude practices and optimistic reframing may counteract catastrophic cognitions that fuel disease-related fear, while the establishment of progressive rehabilitation goals may enhance self-efficacy and mastery beliefs that buffer against fears of physical vulnerability (22).

The particularly pronounced improvements observed in the relationships dimension of the PERMA profile merit specific consideration. Social support has been consistently identified as a protective factor in cardiovascular populations, with meta-analytic evidence demonstrating associations between social isolation and adverse cardiac outcomes (26). The PERMA intervention's explicit focus on strengthening family involvement, facilitating peer support connections, and enhancing therapeutic relationships may have produced synergistic effects that amplified outcomes across multiple well-being dimensions. Patients who developed stronger social connections may have experienced enhanced motivation for rehabilitation engagement, greater emotional resources for coping with recovery challenges, and more effective behavioral modeling and reinforcement from supportive others. These findings align with research emphasizing the importance of addressing social determinants of cardiovascular health and support prioritization of relationship-focused components in cardiac rehabilitation programming.

The observed associations between psychological improvements and physiological cardiac outcomes provide preliminary support for psychophysiological pathways linking positive psychological states to cardiovascular health (23). The intervention group demonstrated superior left ventricular ejection fraction, greater six-minute walk distance, lower BNP levels, and more favorable resting heart rates compared to controls. While the retrospective design precludes definitive causal inference, these patterns are consistent with hypothesized mechanisms through which psychological well-being may influence cardiac physiology (24). Reduced psychological distress may diminish chronic sympathetic nervous system activation, decreasing catecholamine exposure and associated adverse myocardial effects. Enhanced engagement in rehabilitation activities may promote physiological adaptation and cardiovascular conditioning. The meaning and accomplishment dimensions may support sustained motivation for health-promoting behaviors that cumulatively improve cardiac functional status (21). Future prospective research incorporating biomarker assessments and autonomic measures would strengthen mechanistic understanding of these relationships.

The behavioral adherence advantages observed in the intervention group carry substantial implications for long-term cardiovascular secondary prevention (27). Participation in cardiac rehabilitation exercise and dietary modification represent cornerstone elements of post-CABG care that significantly influence outcomes including subsequent cardiovascular events and mortality. The finding that PERMA-based intervention was associated with superior adherence across rehabilitation and dietary domains suggests that psychological well-being enhancement may function as an upstream determinant enabling multiple downstream health behaviors (28). Patients experiencing greater positive emotion, engagement, and sense of accomplishment may possess enhanced self-regulatory capacity and motivation for sustained health behavior change. These findings are consistent with broaden-and-build theory predictions that positive emotional states expand cognitive and behavioral repertoires in ways that facilitate adaptive functioning. The potential for psychological interventions to improve adherence represents an important avenue for addressing the persistent challenge of suboptimal treatment compliance in cardiovascular populations.

The quality of life improvements observed across SF-36 domains underscore the comprehensive functional benefits associated with PERMA-based intervention. Notably, significant advantages were observed in both physical and mental health dimensions, with particularly large effect sizes for mental health, vitality, and general health perceptions (36). These findings are consistent with evidence from cardiac rehabilitation programs demonstrating that comprehensive interventions addressing both physical and psychological dimensions yield superior quality of life outcomes compared to exercise-only approaches (15, 16). The absence of significant differences in bodily pain scores may reflect that pain experience during recovery is predominantly determined by surgical and tissue-healing factors less susceptible to psychological intervention influence. However, the robust improvements in functional domains including physical functioning, role limitations, and social functioning indicate that psychological well-being enhancement translated into meaningful improvements in patients' capacity to engage in daily activities and fulfill valued social roles (29). These findings support the conceptualization of psychological intervention not merely as addressing distress but as promoting positive functional outcomes that enhance overall life quality.

The clinical implications of these findings are substantial for cardiac surgery nursing practice and rehabilitation program development (19). The PERMA model provides a structured, theoretically grounded framework that can be feasibly integrated into existing care pathways. As demonstrated in our implementation, the intervention was delivered by trained nursing staff who completed a 40-hour certification program, suggesting that widespread adoption would not require extensive additional resources or specialized mental health personnel. The intervention components, including gratitude documentation, goal-setting, social support facilitation, and meaning exploration, can be implemented within routine clinical encounters across in-hospital, transitional, and maintenance phases. The evidence that such intervention is associated with measurable improvements in both psychological and physiological outcomes strengthens the case for reconceptualizing cardiac rehabilitation as a truly comprehensive biopsychosocial endeavor rather than a primarily exercise-focused program. Healthcare systems seeking to optimize post-surgical outcomes should consider systematic implementation of positive psychological intervention protocols as a core component of cardiac care.

Several methodological limitations warrant acknowledgment when interpreting these findings. First, the retrospective cohort design, while offering ecological validity and practical feasibility, cannot establish causal relationships with the certainty achievable through randomized controlled trials (14). Although group allocation was determined by nursing team assignment based on ward scheduling rather than patient characteristics, and both unadjusted and multivariable-adjusted analyses yielded consistent results, residual confounding from unmeasured variables cannot be excluded. Selection bias may have influenced group composition, as patients receiving PERMA intervention may have differed systematically from controls in unmeasured characteristics such as baseline psychological resilience, family support, or motivation levels that could influence outcomes. Second, the single-center design limits generalizability to other clinical settings and patient populations, and the specific cultural context of Chinese cardiac patients may affect the transferability of findings to other healthcare systems. Third, the three-month follow-up duration, while capturing meaningful short-term outcomes, does not permit assessment of intervention durability or effects on longer-term cardiovascular events such as graft patency or major adverse cardiac events. Fourth, the reliance on self-report measures for psychological outcomes and behavioral adherence introduces potential for response bias, social desirability effects, and recall inaccuracies, though the consistency of findings across multiple instruments and the inclusion of objective physiological measures provide some reassurance regarding measurement validity. Fifth, the multiplicity of tested endpoints increases the risk of Type I error; while the primary outcomes of FoP-Q-SF and PERMA overall well-being consistently demonstrated significant associations, secondary outcomes should be interpreted with appropriate caution. Sixth, we did not incorporate formal assessment of intervention fidelity using independent raters, although structured documentation templates and regular team meetings provided some quality assurance. Future research should employ prospective randomized designs with extended follow-up periods, formal fidelity assessment, and multisite recruitment to more definitively establish intervention efficacy and long-term benefits.

In conclusion, this retrospective cohort study provides substantial evidence supporting the association between PERMA model-based positive psychological intervention and reduced fear of disease progression and enhanced subjective well-being among patients recovering from coronary artery bypass grafting surgery (5). The intervention was associated with improvements across multiple outcome domains including psychological distress, well-being, cardiac function, behavioral adherence, and quality of life. These findings contribute to the growing evidence base supporting integration of positive psychology principles into cardiac care and provide practical guidance for clinical implementation. Continued research through prospective randomized controlled trials is warranted to confirm causal relationships, elucidate underlying mechanisms, and optimize intervention delivery for diverse patient populations.

## Data Availability

The original contributions presented in the study are included in the article/Supplementary Material, further inquiries can be directed to the corresponding author.
